# A metagenomic assessment of bacterial community in spices sold open-air markets in Saint-Louis, Senegal

**DOI:** 10.1038/s41598-024-65756-0

**Published:** 2024-06-26

**Authors:** Sarbanding Sané, Abou Abdallah Malick Diouara, Seynabou Coundoul, Sophie Déli Tene, Alé Kane, Serigne Fallou Wade, Abdoulaye Tamba, Mamadou Diop, Mame Ndew Mbaye, Fatou Thiam, Modou Dieng, Malick Mbengue, Cheikh Momar Nguer, Aminata Diassé Sarr, Ababacar Sadikh Ndao, Coumba Touré Kane

**Affiliations:** 1 Groupe de Recherche Biotechnologies Appliquées & Bioprocédés Environnementaux (GRBA-BE), École Supérieure Polytechnique (ESP), Université Cheikh Anta DIOP, 5085 Dakar-Fann, Dakar, Senegal; 2 Laboratoire des Sciences Biologiques, Agronomiques, Alimentaires et de Modélisation des Systèmes Complexes (LABAAM), UFR S2ATA, Université Gaston Berger, 234, Saint-Louis, Senegal; 3 École Supérieure des Sciences Agricoles et de l’Alimentation, Université Amadou Makhtar MBOW, Dakar, Senegal; 4 Institut Supérieur d’Enseignement Professionnel (ISEP), Bignona, Senegal; 5 Laboratoire d’Analyses et Essais (LAE), École Supérieure Polytechnique (ESP), Université Cheikh Anta DIOP, 5085 Dakar-Fann, Dakar, Senegal; 6 Laboratoire de Microbiologie Appliquée et de Génie Industriel, École Supérieure Polytechnique (ESP), Université Cheikh Anta Diop, 5085 Dakar-Fann, Dakar, Senegal; 7 Institut Supérieur d’Enseignement Professionnel (ISEP), Matam, Senegal; 8 Institut de Technologie Nucléaire Appliqué (ITNA), Université Cheikh Anta DIOP, 5005 Dakar, Senegal; 9 Institut de Technologie Nucléaire Appliqué (ITNA), Université Cheikh Anta DIOP, 5005 Dakar, Senegal; 10 Université Sine Saloum El Hadj Ibrahima Niass (USSEIN), Kaolack 55, Senegal

**Keywords:** Spices, Metagenomic, Bacterial community, Sequencing, Bacteria, Microbial communities, Pathogens

## Abstract

Natural spices play an essential role in human nutrition and well-being. However, their processing on different scales can expose them to potential sources of contamination. This study aimed to describe the bacterial community genomic footprint in spices sold in Senegal. Spice samples were collected in August 2022 in Saint-Louis, Senegal. The genomic region coding bacterial 16S rRNA was then amplified and sequenced using Oxford Nanopore Technology (ONT). Sequencing was carried out on two batches of samples, one containing part of the “Local Spices or Herbs” (n = 10), and the other, a mixture of 7 spices, Curcuma, Thyme and the other part of the “Local Spices or Herbs” (n = 39). Results showed high bacterial diversity and the predominance of *Escherichia coli* and *Salmonella enterica* in samples, with total reads of 65,744 and 165,325 for the two batches, respectively. The sample category “Homemade mixture of food condiments “, which includes all “Local Spices or Herbs” samples, showed remarkable bacterial diversity. These were followed by Curcuma, a blend of 7 spices and thyme. Also, the different categories of spices studied show similarities in their bacterial composition. These results highlight the microbial community’s highly diverse genomic profile, including pathogenic bacteria, in spice samples.

## Introduction

Spices have been used for centuries as an essential component in traditional Asian medicine^1,2^. Some of them draw special attention due to their use in the cosmetics and pharmaceutical industries^3^. Spices are reputed to be a source of bioactive compounds^4^ with sometimes rich extracts in polyphenolic compounds, including high levels of antimicrobial, antioxidant and anti-inflammatory activity^5,6^. Preventive and curative actions of spices against cancer, gastrointestinal disorders, psychological disorders, diabetes, obesity, metabolic syndromes and neurological disorders were also reported^7,8^. Aromatic plants and spices have thus become well established over time, justifying their use by around 80% of the world’s population, mainly in developing countries^7^. Spices can be divided into several groups: strong spices (pepper, chilli, ginger), mild spices (paprika, coriander), aromatic spices (cinnamon, curcuma, cloves, cumin, anise, celery), dried herbs (basil, bay leaves, dill, marjoram, tarragon, thyme) and aromatic vegetables, for instance, onions or garlic^9^.

From a nutritional point of view, China and some West African countries have begun to use condiments and seasonings to remedy significant dietary deficiencies, particularly in iodine, iron and vitamin A^10^. According to several studies, various micronutrients are added to condiments depending on the deficiencies demonstrated in the target population. The bioavailability of the micronutrient remains highly important, as it must be absorbed by the body in sufficient quantities to contribute effectively to improving these deficiencies^10,11^. Despite the awareness messages to limit the overconsumption of processed food products while promoting natural products in the diet^12–14^, the use of industrial ingredients remains a concern^15^. However, spices and broths made from natural products can also be at risk for consumers. Indeed, studies have reported that, due to transformation processes such as extraction and packaging, these products can be contaminated by bacteria such as *Salmonella *spp, *Escherichia coli*, *Bacillus *spp, *Staphylococcus *spp, as well as viruses, parasites and pathogenic fungi during handling^16–18^. They can, therefore, have a very varied diversity, including Aerobic bacteria and Spore-forming bacteria, which can survive for a long time in spices^19^. Furthermore, the diversity and abundance of microbial communities in these food matrices can influence their compositions, nutritional benefits and even the functioning of the gut ecosystem^20–22^. The ability of microorganisms in spices to survive and persist has led to a higher incidence of foodborne illnesses and outbreaks, mainly in developing countries. Indeed, studies have reported that among foods imported into the United States between 1996 and 2014, herbs and spices accounted for 1.5% of all foodborne illnesses linked to foods with low water activity. Also, 14 spice-associated foodborne outbreaks between 1973 and 2010 in Canada, Denmark, England and Wales, France, Germany, New Zealand, Norway, Serbia and the United States resulted in 2 deaths, 128 hospitalizations and 1946 illnesses were documented. Infants and children were most affected by these outbreaks (36% of cases). Salmonella was the primary agent responsible for 71% of outbreaks and 87% of illnesses; Bacillus spp. was responsible for 29% of outbreaks and 13% of illnesses^23,24^.

Traditional studies of bacterial diversity in foods have relied on culture-dependent methods, whereas only around 1% of microorganisms present in the natural environment can be grown by these methods^25,26^. Increasingly, molecular biology approaches are being used such as gradient gel electrophoresis (DGGE) and polymerase chain reaction (PCR) and extend later to metagenomic methods used to assess microbial diversity and analyse microbial community structures in diverse environments including food matrices^26–29^. This study used the sequencing method to describe the genomic footprint of the microbial community present in spices sold in open-air markets in Saint-Louis, Senegal. Two main categories of spices were used: “Homemade mixture of food condiments” and “Pseudo-industrial spices.” The former includes “Local Spices or Herbs,” which are fresh spices, while the latter consists of dried spices such as turmeric, 7-spice mix, and thyme.

## Results

A total of 49 samples were analysed in this study. The “Homemade mixture of food condiments (ready to use)” category comprises 19 samples of “Local Spices or Herbs”, while the “Pseudo-industrial spices” category comprises a total of 30 samples. Local Spices or Herbs” are a mixture of mainly green onion (*Allium fistulosum*), garlic (*Allium sativum*), green bell pepper (*Capsicum annuum *L.) and fresh chilli pepper. The first batch of samples analysed consisted of 10 samples of “Local Spices or Herbs” and the second batch of turmeric, a mixture of 7 spices, thyme (10 samples each) and nine samples of “Local Spices or Herbs”. For each sample type, further details, such as numbers and collection locations, are given in Table 1.

**Table 1:** Distribution of samples according to type and collection sites.

Categories	Types of samples	Dry or dehydrated form	Number of collected specimens	Collection sites	Data set 1	Data set 2
“Homemade mixture of food condiments (ready to use)”	Local spices or herbs	no	19	Sor/Saint-Louis	**x**	**x**
Pseudo-industrial spices	Mixture of 7 spices	yes	10	Sor, Guet Ndar and Pikine/Saint-Louis		**x**
	Curcuma	yes	10	Sor, Guet Ndar and Pikine/Saint-Louis		**x**
	Thyme	yes	10	Sor, Guet Ndar and Pikine/Saint-Louis		**x**

The samples are of different types and were collected from different markets in Saint-Louis. The “categories” column highlights the difference between dehydrated spices, such as curcuma, thyme and 7-spice mixture, and non-dehydrated spices, such as Local Spices or Herbs.

**Figure 1:**
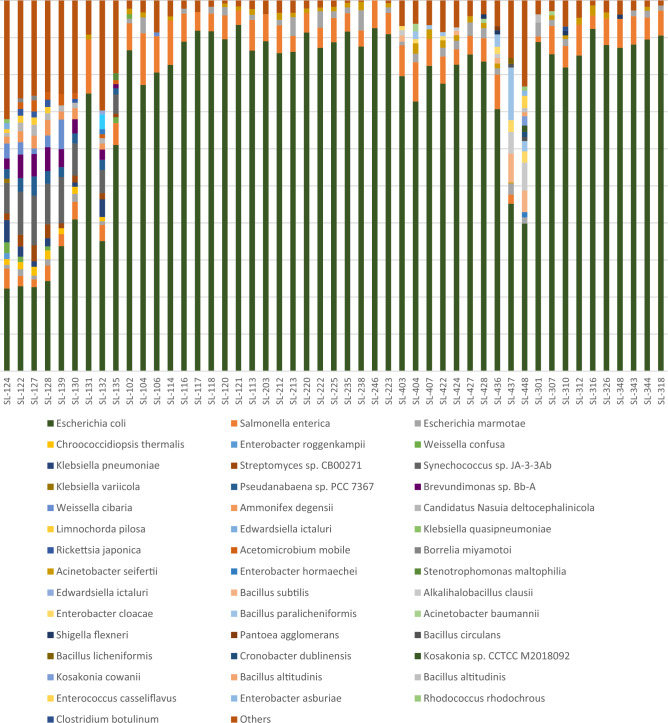
Relative distribution of bacterial species in samples. This figure was produced using Microsoft Excel 2016. It describes the relative abundance of bacterial species in each spice sample studied. The “others” section contains bacterial with less than 1% abundance.

Sequencing data were processed using Nanopore's Epi2me software (version 3.6.1). Quality scores were 9.87 and 9.82, with classified read counts of 65,744 and 165,325, respectively for the two batches analyzed. Figure 1 shows the predominant bacterial species for all the samples processed in this study. Generally speaking, *Escherichia coli* and *Salmonella enterica* are the most represented species in all samples (Fig. 1).

Analysis of bacterial diversity according to spice type showed the predominance of *Escherichia coli*, *Salmonella enterica* and *Acinetobacter seifertii*, with proportions differing slightly from one category to another (Supplement 1–4, Supplementary Information). All species with an abundance of less than 1% were merged into an “others” category. This group represented 21.2% of species in the “Local Spices or Herbs” and was mainly made up *of Saccharothrix sp.6-C* (4.7%), *Shigella flexneri* (4.5%) and *Buchnera aphidicola* (4.1%). For Curcuma, the “others” group represented 9.1% and was mainly made up of *Acinetobacter baumannii* (6.1%), *Bacillus circulans* 3.9% and *Kosakonia cowanii* (3%). For the 7 spices and Thyme samples, this group represented 4% and 2%, respectively. The predominant bacterial species were *Acinetobacter baumannii* (16.3%), *Clostridium botulinium* (11.3%) and *Clostridium tatani* (3.6%) for 7 spices, *and Shigella flexneri* (12.4%) and *Pseudomonas fulva* (6.7%) for Thyme. These relative abundances were calculated based on the total number of species in the “other” category.

**Figure 2:**
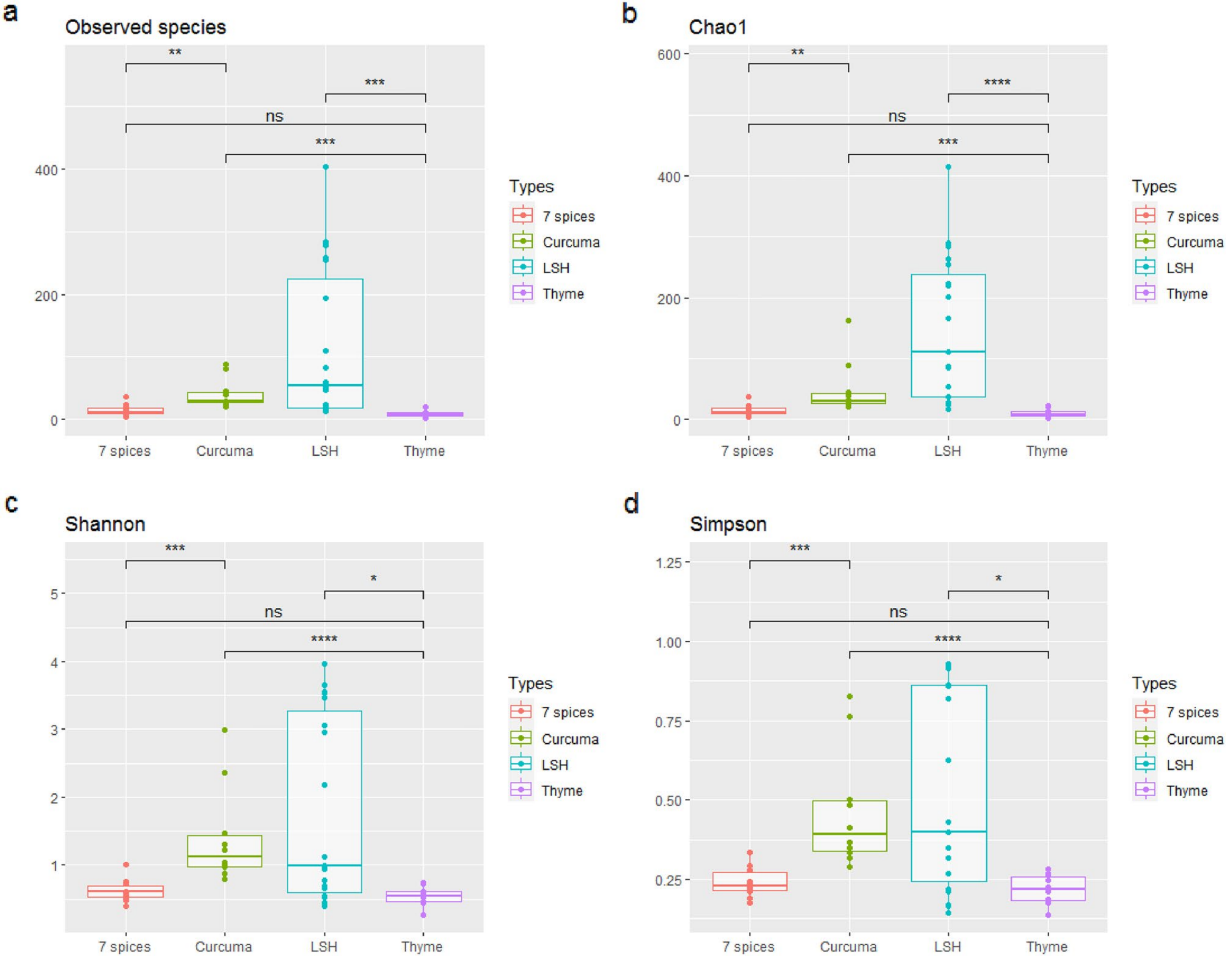
Alpha-diversity analysis. This figure was produced using R software version 4.1.3 to observe alpha-diversity, representing the diversity of bacterial species within a single spice group such as Curcuma, 7 spice, local spices or thyme. This alpha diversity is observed through its different parameters: the “observed species” (Fig. 2a) gives the number of bacterial species observed in each spice group considered; the chao1 index (Fig. 2b) gives the actual number of bacterial species in the environment based on the previously estimated number; the Shannon index (Fig. 2c), which shows the main differences in the diversity of the spice groups studied, as the higher the index, the greater the diversity; the Simpson index (Fig. 2d), which completes the interpretation of alpha diversity, as it varies between 0 and 1, showing maximum diversity if it tends towards 1 and minimum if it tends towards 0.

By considering all the types of spices, alpha-diversity analysis showed that “Local Spices or Herbs” had the most incredible diversity, with an average number of taxa per sample of 115.78. This group is followed by Curcuma, a mixture of 7 spices and Thyme, respectively. The average taxa numbers per sample were 41.1, 14.5 and 10.3, respectively (Fig. 2). About the number of species per spice group, after using the Wilcoxon test, there was a significant difference between “Local Spices or Herbs” and Thyme (*p* < 0.001), Curcuma and Thyme (*p* < 0.001), and Curcuma and the mixture of 7-spice (*p* < 0.01). However, there was no statistically significant difference between the mixture of 7-spice and Thyme, as shown in Fig. 2A and B. Figure 2C and D show that species' relative abundance and stability are greater for “Local Spices or Herbs”. This group shows a significant difference with Thyme (*p* < 0.05). There is also a significant difference between Curcuma and Thyme (*p* < 0.001) and between Curcuma and the mixture of 7-spice (*p* < 0.001).

**Figure 3:**
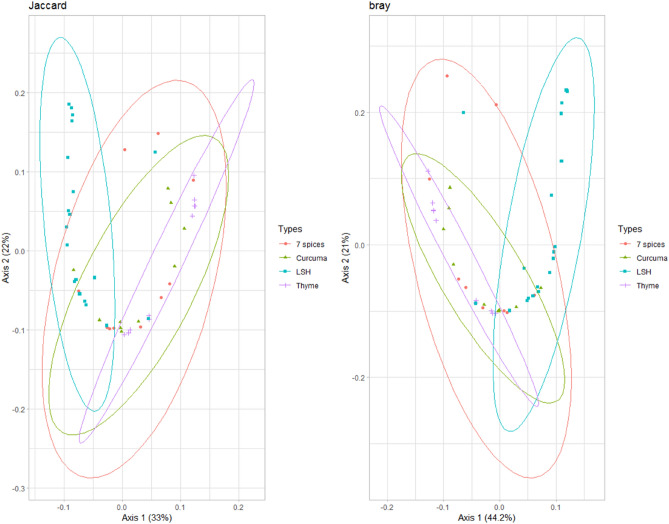
Beta-diversity analysis. This figure was produced using R software version 4.1.3. The aim is to compare bacterial diversity in the different spice groups studied using Jaccard and Bray Curtis distances.

**Figure 4:**
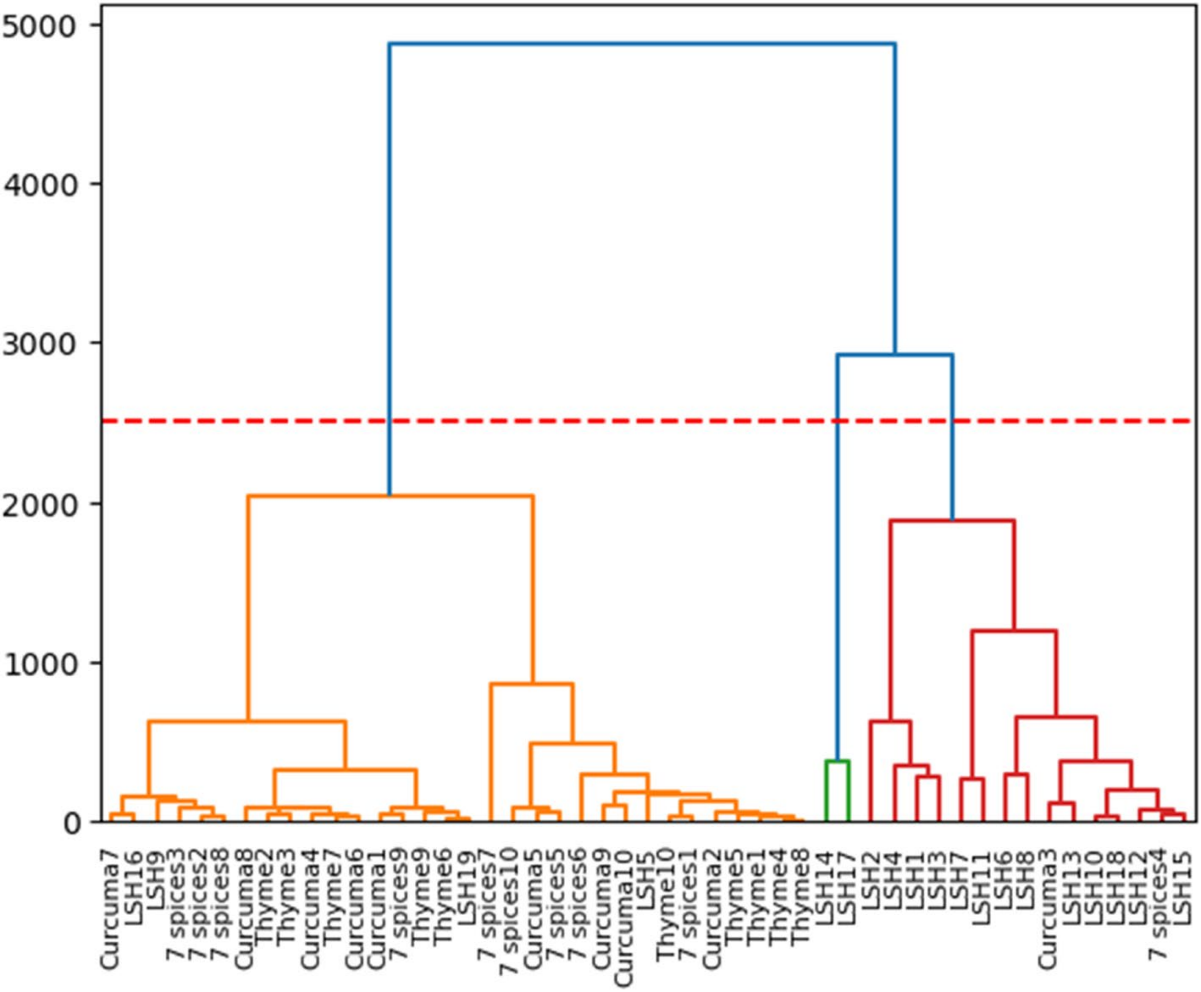
Hierarchical clustering of samples.

Beta-diversity was illustrated using PcoA based on Bray–Curtis dissimilarity and Jaccard distance, which showed similarities in bacterial composition between samples from the different spice groups and showed two clusters (Fig. 3). One consisted of the species found in Thyme, Curcuma, a mixture of 7 spices samples and part of the “Local Spices or Herbs” samples. The second cluster consists solely of “Local Spices or Herbs” samples. These results show that the bacterial communities in the samples from the first cluster are closer than those in the second cluster. The PCoA values obtained are 33% (axis1) and 22% (axis2) for the Jaccard distance and 44.2% (axis1) and 21% (axis2) for the Bray–Curtis index. Similarly, hierarchical clustering mainly shows two clusters formed by the spices studied (Fig. 4). The first is made up of thyme, turmeric, and 7-spice blend samples. The second cluster is mainly formed by LSH samples.

## Discussion

This study aimed to describe the microbial community present in spices sold in open-air markets in Saint-Louis using the sequencing method. The Oxford Nanopore Technology (ONT) sequencing method used in this study allowed us to detect a broad-spectrum genomic footprint of micro-organisms, including those usually not detected by culture-dependent methods. Indeed, since the revolutionary improvement in DNA sequencing technologies, direct high-throughput analysis of the genomic DNA of an entire community without prior culture has become the most common approach, overcoming the constraints of conventional microbiological approaches^30^. Several technologies have been developed over the years to achieve this. However, the Oxford Nanopore Technology (ONT) sequencing method stands out because of its advantages, notably the production of long sequence reading fragments. Additionally, this technology also offers real-time analysis during sequencing. It is even more flexible regarding the number of samples to be analysed simultaneously, including portability, all at a lower cost than other sequencing technologies. From a technical point of view, the preparation time of the sequencing library is less restrictive, with quantities of necessary DNA sometimes being reduced compared to other techniques^31^. In addition, from a technological point of view, MinION sequencing is mainly used to address metataxonomy^32^.

Our results show the presence of many pathogenic bacteria in the spice samples studied, with their abundance varying according to the type considered. Studies in countries like Brazil have reported similar results^33^. In contrast to other studies in Vietnam, Iran, Indonesia, India, and the Netherlands, analyzed spices meet established microbiological standards and were of satisfactory quality^35,36^. These were particularly spices, herbs, and dried spices produced on a small scale.

The significant bacterial communities found in the “Local Spices or Herbs” samples analyzed compared to the others could be explained by the difference in water activity. The “Local Spices or Herbs” samples, consisting of a mixture of aromatic herbs and food condiments, represent a more favorable environment for bacterial growth than the other dried spices. Nevertheless, despite their packaging giving them “stable physicochemical characteristics”, the dehydrated products, such as Curcuma, the mixture of 7-spice and thyme, have a relatively large bacterial community (Supplementary information). These foods generally undergo drying processes at ambient temperature at the place of production. Moreover, in the developing countries from which they originate, harvesting and production technologies do not necessarily comply with optimal sanitary conditions^32–34^. The non-compliance with hygienic measures at all stages of the process, particularly during agricultural production, harvesting, washing and sun-drying, may explain the results of this study. Packaging hygiene and storage conditions are not always respected either, which is a probable cause of food contamination (FAO, https://www.fao.org/3/w7429f/w7429f0r.htm). All these factors lead to high levels of microbial contamination, which means these products may not be suitable for human consumption^32,35,36^.

The presence of *Escherichia coli*, *Salmonella *spp. and *Bacillus *spp. in most of the analyzed samples could potentially be harmful in herbs and in various food matrices such as spices, which were the subject of this study^39^. Moreover, the bacterial spores introduced by spices can withstand various preparation processes, including heat treatment^40^. The high presence of *Escherichia coli* in all samples may indicate faecal contamination of various origins. In the case of fat-in-house spices, including horticultural products, faecal contamination could be irrigation water from wastewater systems used without prior biological treatment^40–45^. As for the other spices studied, faecal contamination could be due to a hygiene failure by operators handling the processing (reduction to powder, mincing, etc.), storage and transport stages.

The analysis of distance matrices containing dissimilarity information can effectively capture significant and subtle compositional differences between the samples studied^47^. Other techniques, such as the Unweighted Pair Group Method with Arithmetic Mean (UPGMA), an agglomerative hierarchical clustering method widely used in practice, can also be considered. Step by step, it combines the two closest groups or elements into a higher-level group, and the distance between the new group and any other group is calculated as the arithmetic mean distance between the elements in the different groups or clusters^48,49^.

The small sample size (n = 49) in this study constitutes a limitation. Furthermore, the presence of genetic material cannot provide information on the level of bacteriological contamination. Therefore, the description of the microbial community obtained by the metagenomic approach could have been accompanied by conventional microbiological analyses to highlight the fraction of viable bacteria in the analyzed samples.

## Methods

### Collection and preparation of samples

Samples of Curcuma, 7-spice mixture (clove, ginger, garlic, cumin, lemon, nutmeg and salt), Thyme and “Local Spices or Herbs” (a mixture of food condiments used to season dishes) were collected from 3 Saint-Louis markets in August 2022, in line with standards for sampling food matrices in general and spices in particular^50^. For dry spices such as turmeric, 7-spice mixture and thyme, 30 g was purchased, compared with 100 g for “Local Spices or Herbs”. Each sample purchased was put in a sterile zipped bag. Presented in dehydrated form and sold in original packaging, the pseudo-industrial spices (Curcuma, 7-spice blend and thyme) were transported and stored at room temperature until use. On the other hand, the “Local Spices or Herbs” samples were placed and transported in an isothermal bag maintained at +4 °C to the laboratory within 4 h of purchase, thus ensuring their preservation. Samples were then stored at − 20 °C until use. A total of 49 samples divided into two categories, including “Homemade mixture of food condiments (ready to use)” (n = 19) and “pseudo-industrial spices” sold dehydrated with primary packaging (n = 30), were collected and analyzed in two batches. The first batch consists of 10 “Local or Spices Herbs” samples. This type of spice is a blend of green onions, peppers, garlic and Sofia bell pepper (*Capsicum annum*). The second batch, designated as “pseudo-industrial spices,” was made up of thyme, 7-spice mix, Curcuma, and the “Local or Spices Herbs” samples. For each sample type, additional details, such as the numbers and collection locations, are reported in Table 1.

### Extraction of Bacterial DNA

Samples were subjected to manual extraction using the *Omega E.N.Z.A Food DNA kit *(Omega Bio-Tek, GA, USA) dedicated to food matrices that are particularly difficult to lyse with a few minor modifications. To optimize cell lysis, four beads with a diameter of 2.0 mm were added to the lysis buffer before beating the sample with Analog Disruptor Genie during 5 min. These ultra-high-density beads are fracture-resistant, chemically inert and ideal for disrupting difficult-to-analyse biological samples. The DNA concentration was determined by a Nanodrop spectrophotometer One (Thermo Scientific, USA), and the sample was stored at − 20 °C until analysis.

### PCR Amplification and Sequencing

The region coding for the 16S subunit of bacterial RNA was amplified using the universal primers Fd1 (5′ – AGA GTT TGA TCM TGG CTC AG – 3′) and Rd1 (5′ – AAG GAG GTG ATC CAG CC– 3′)^51^. The reaction volume for amplification was 25 μL and included 12.5 μL of *One Taq*^*®*^* Quick-Load 2X Master Mix with Standard Buffer* kit (New England Biolabs, M0486), 2 μL of primers, 5 μL of DNA and 5.5 μL of molecular grade water. Polymerase Chain Reaction was performed under the following conditions: an initial denaturation of 94 °C for 30 s followed by 30 cycles of 94 °C for 30 s, 50 °C for 30 s, 68 °C for 1 min and a final extension of 68 °C for 5 min. Amplified fragments were visualized by electrophoresis on a 1% agarose gel using ethidium bromide staining and UV light. Amplicons were then purified using the Zymo DNA clean & concentrator ™ kit-5 (Zymo D4034). Library preparation was performed using the Rapid Barcoding Kit (SQK-RBK 110-96). The sequencing was performed on Flow Cell R9.4.1 (FLO-MIN106D) on a MinIon Mk1C sequencer. Dorado, MinKNOW's integrated production basecaller, uses a bidirectional recurrent neural network in which information can be passed from one node to another to perform the base call live during the sequencing cycle. Basecalling algorithms process the signal stored in POD5 files to decode the sequence of bases into FASTQ files. MinKNOW version 23.04.5 is used in this study, with a minimum FastQ file quality score of 7. A real-time workflow Epi2me platform (version 3.6.1) was used for species identification. Data go through the following processing stages: FASTQ files are produced by the MinKNOW software after sequencing with the MinION and then downloaded by Epi2me (version 3.6.1). The basecalling reports are updated, and reads meeting the quality threshold are sent to the classification stage. In this classification step, a pre-constructed database based on NCBI taxonomy and a reference genomic sequence database (RefSeq) is used. After this processing, the species are classified based on the number of reads for each sequenced sample. Alpha diversity, which shows the complexity of species within a sample, was obtained using four indices: Chao1, Shannon, Simpson and observed species. For this, the Vegan and Phyloseq packages of the R Studio software were used. Beta diversity, which shows the complexity of species across all samples, was measured using Jaccard and Bray Curtis indices. To obtain this diversity, the Vegan and Ecodist packages of the R software were used. All figures were also generated using the R software. The newly generated sequences have been deposited in Genbank (https://www.ncbi.nlm.nih.gov/genbank/) and are available under the following accession numbers: SAMN41665108 to SAMN41665156.

### Statistical analysis

All statistical analysis was performed on R software version 4.1.3 available on http://cran.r-project.org/bin/windows/base/. For each considered spice group, the Wilcoxon test was performed for differential analysis of diversity. The significance level was set at *p* < 0.05. The degree of significance is proportional to the number of stars shown at the top of the figures (* corresponds to *p* < 0.05, ** corresponds to *p* < 0.01 and *** corresponds to *p* < 0.001). In order to assess the similarity between the different microbial communities in the considered spice groups, beta-diversity was calculated based on the Bray–Curtis dissimilarity distance and the Jaccard index. The result was then represented using a principal coordinate analysis (PCoA).

### Statement

The collection of spice samples (including plant fragments or extracts) and all experiments in this study complied with relevant institutional, national, and international guidelines and legislation.

## Conclusion

This study aimed to describe the bacterial community in spices sold in open-air markets in Saint-Louis, Senegal. It revealed that spices, whether fresh (“Local Spices or Herbs”) or dehydrated (Curcuma, Thyme, a mixture of 7 spice), can carry bacteria, which could be pathogens such as *Salmonella *spp, *Shigella *spp, *Bacillus *spp, *Enterobacter *spp, *Pseudomonas *spp, *Klebsiella *spp. In addition, it highlighted potential defective hygiene in the production chain of these spices through the presence of indicators of faecal contamination such as *Escherichia coli* and *Salmonella enterica* species. These results suggest that efforts must be made to raise awareness among the various stakeholder in the production chain. In addition, further research with a more significant number of samples of spices should be carried out to identify other potential contaminants that could harm to human health.

### Supplementary Information

Supplementary Information.

## Data Availability

The newly generated sequences have been deposited in Genbank (https://www.ncbi.nlm.nih.gov/genbank/) and are available under the following accession numbers: SAMN41665108 to SAMN41665156.

## Abbreviations

DGGE: Gradient gel electrophoresis
DNA: Deoxyribonucleic acid
FAO: Food and Agriculture Organization of the United Nations
GRBA-BE: Applied Biotechnologies and Environmental Bioprocesses Research Group
IRESSEF: Institute for Health Research, Epidemiological Surveillance and Training
ISEP: Higher Institute for Vocational Training
ITNA: Institute of Applied Nuclear Technology
LABAAM: Biological, Agronomic, Food Sciences and Complex Systems Modelling Laboratory
LAE: Analysis and Testing Laboratory
LSH: Local spices or herbs
ONT: Oxford Nanopore Technology
PCoA: Principal coordinate analysis
PCR: Polymerase chain reaction
RNA: Ribonucleic acid
UPGMA: Unweighted pair group method with arithmetic mean
USSEIN: Sine Saloum El Hadj Ibrahima Niass University
