# Supplementary material for: A metagenomic assessment of bacterial community in spices sold open-air markets in Saint-Louis, Senegal
Source: Sci Rep. 2024 Jun 26;14:14709. doi: 10.1038/s41598-024-65756-0 (PMC11208442; doi:10.1038/s41598-024-65756-0)
Supplement: Supplementary file 1 — Supplementary Information. [file 41598_2024_65756_MOESM1_ESM.pptx]

## Slide 1
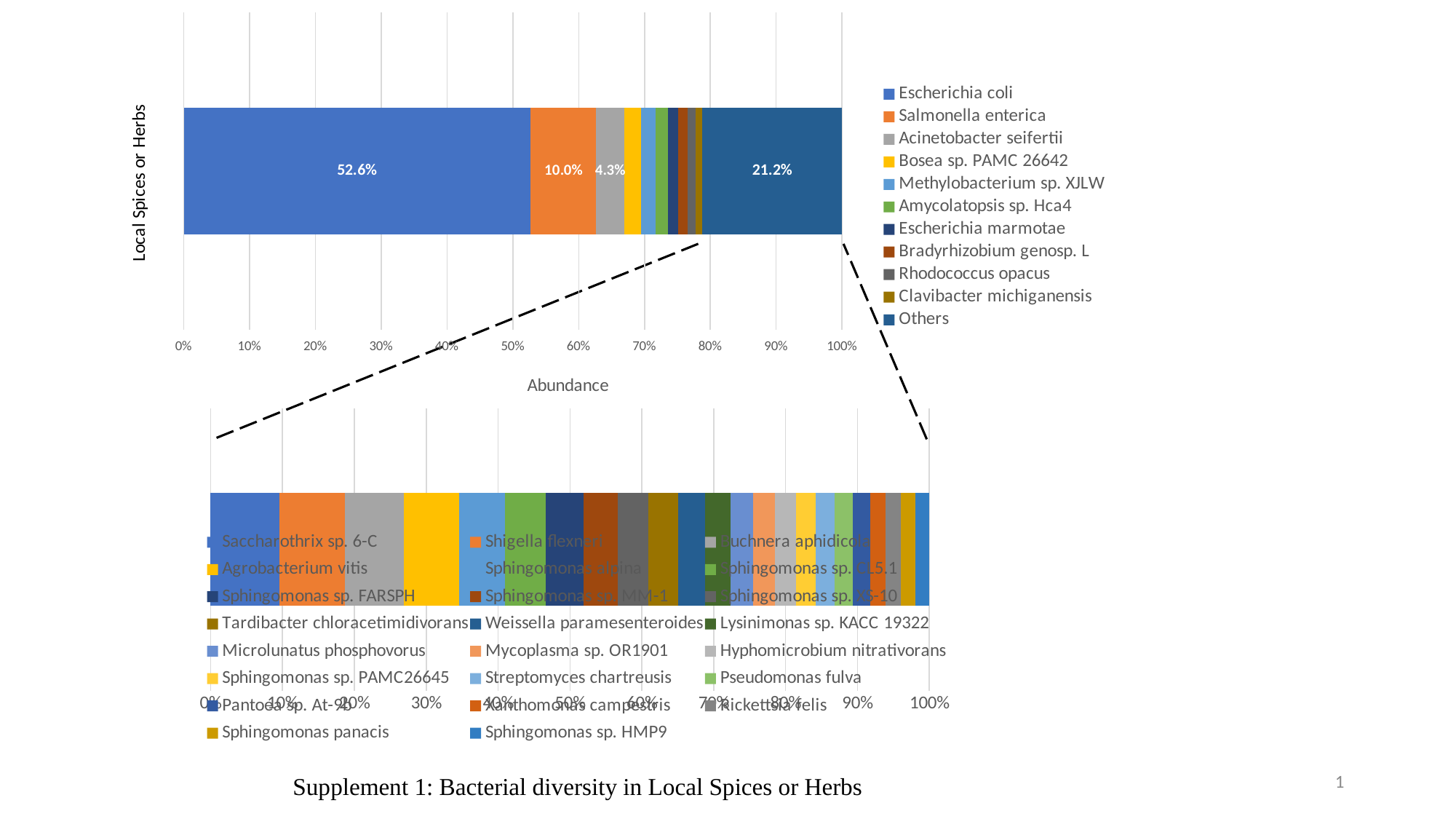

### Chart
| Category | Escherichia coli | Salmonella enterica | Acinetobacter seifertii | Bosea sp. PAMC 26642 | Methylobacterium sp. XJLW | Amycolatopsis sp. Hca4 | Escherichia marmotae | Bradyrhizobium genosp. L | Rhodococcus opacus | Clavibacter michiganensis | Others |
|---|---|---|---|---|---|---|---|---|---|---|---|
### Chart
| Category | | | | | | | | | | | | | | | | | | | | | | | |
|---|---|---|---|---|---|---|---|---|---|---|---|---|---|---|---|---|---|---|---|---|---|---|---|
| 1 | 0.0474076872225895 | 0.0449523089999056 | 0.0408914911700822 | 0.0380583624516007 | 0.031447728775144 | 0.0277646614411181 | 0.0261592218339787 | 0.0237982812352441 | 0.0211540277646614 | 0.0203985267730664 | 0.0186986495419775 | 0.017093209934838 | 0.0159599584474455 | 0.0149211445840023 | 0.0144489564642554 | 0.0135045802247615 | 0.013126829728964 | 0.0124657663613184 | 0.0118047029936727 | 0.0109547643781282 | 0.0101992633865332 | 0.0100103881386344 | 0.00972707526678629 |1
Supplement 1: Bacterial diversity in Local Spices or Herbs

## Slide 2
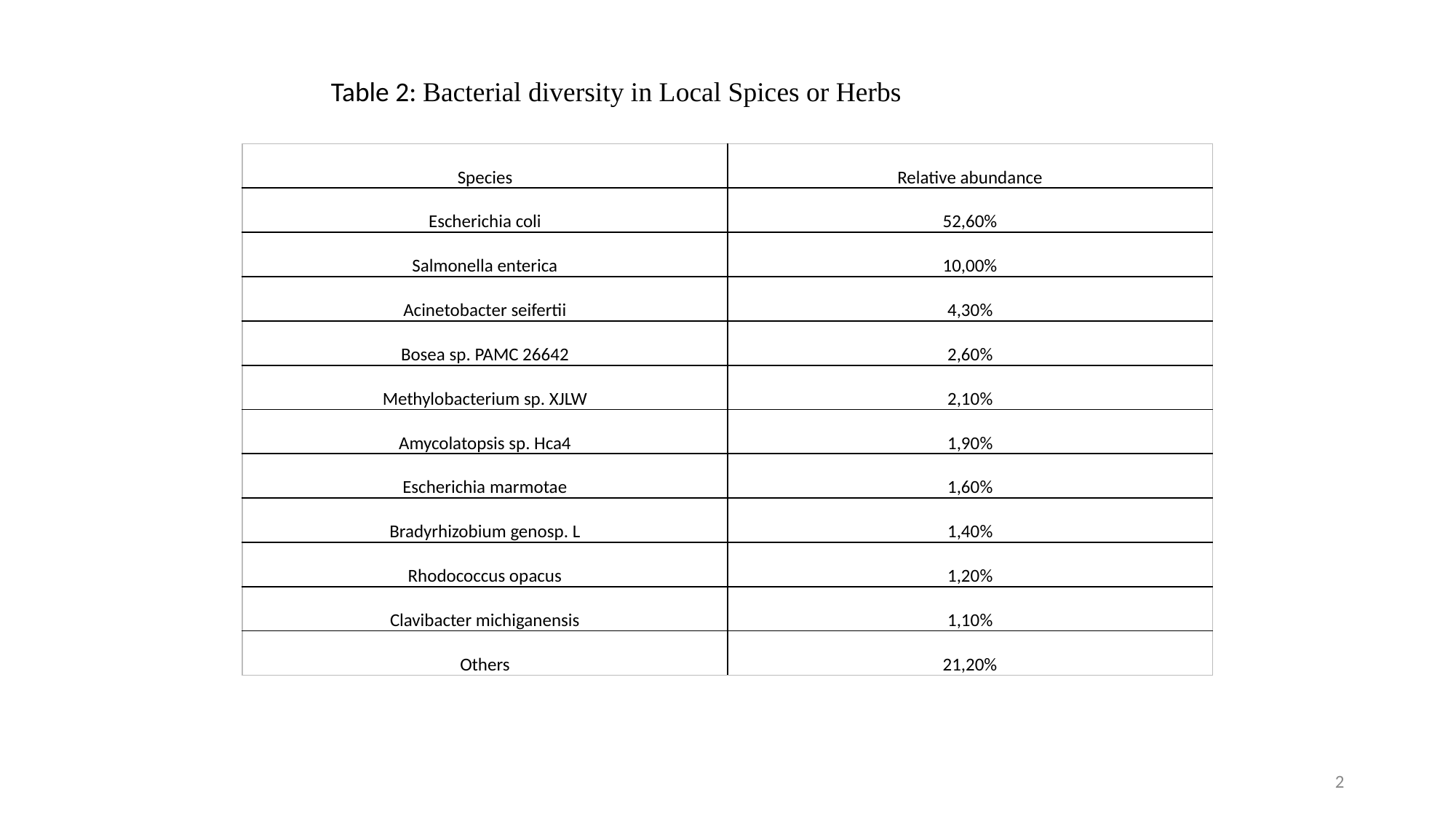

Table 2: Bacterial diversity in Local Spices or Herbs
| Species | Relative abundance |
| --- | --- |
| Escherichia coli | 52,60% |
| Salmonella enterica | 10,00% |
| Acinetobacter seifertii | 4,30% |
| Bosea sp. PAMC 26642 | 2,60% |
| Methylobacterium sp. XJLW | 2,10% |
| Amycolatopsis sp. Hca4 | 1,90% |
| Escherichia marmotae | 1,60% |
| Bradyrhizobium genosp. L | 1,40% |
| Rhodococcus opacus | 1,20% |
| Clavibacter michiganensis | 1,10% |
| Others | 21,20% |
2

## Slide 3
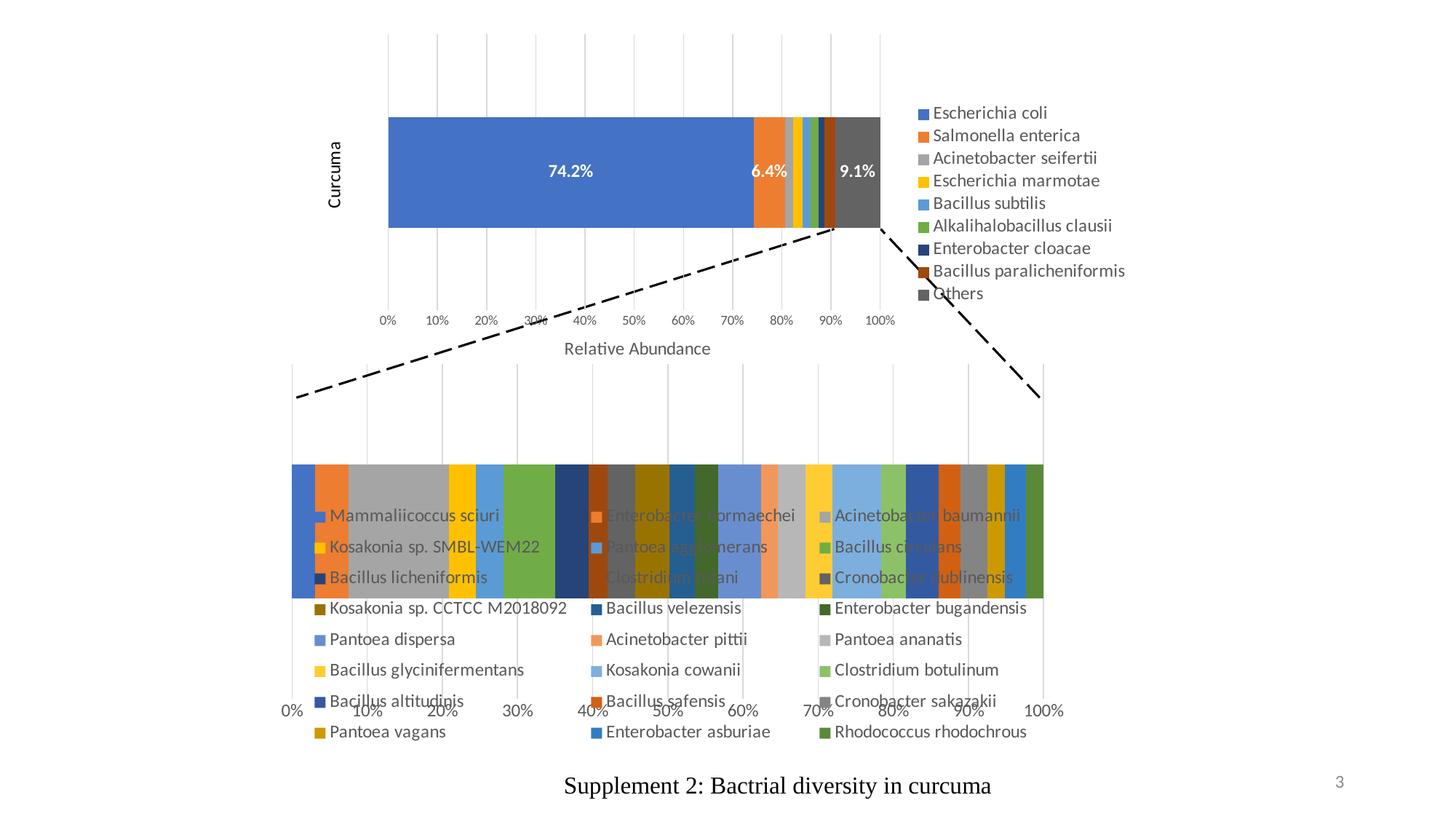

### Chart
| Category | Escherichia coli | Salmonella enterica | Acinetobacter seifertii | Escherichia marmotae | Bacillus subtilis | Alkalihalobacillus clausii | Enterobacter cloacae | Bacillus paralicheniformis | Others |
|---|---|---|---|---|---|---|---|---|---|
| Abundance | 0.7424096127024997 | 0.06383435253728141 | 0.01573389651531151 | 0.019535374868004225 | 0.016736401673640166 | 0.01562829989440338 | 0.01129883843717001 | 0.02270327349524815 | 0.0911 |
### Chart
| Category | Mammaliicoccus sciuri | Enterobacter hormaechei | Acinetobacter baumannii | Kosakonia sp. SMBL-WEM22 | Pantoea agglomerans | Bacillus circulans | Bacillus licheniformis | Clostridium tetani | Cronobacter dublinensis | Kosakonia sp. CCTCC M2018092 | Bacillus velezensis | Enterobacter bugandensis | Pantoea dispersa | Acinetobacter pittii | Pantoea ananatis | Bacillus glycinifermentans | Kosakonia cowanii | Clostridium botulinum | Bacillus altitudinis | Bacillus safensis | Cronobacter sakazakii | Pantoea vagans | Enterobacter asburiae | Rhodococcus rhodochrous |
|---|---|---|---|---|---|---|---|---|---|---|---|---|---|---|---|---|---|---|---|---|---|---|---|---|
| 3.9007092198581603E-2 | 0.0141843971631206 | 0.0200945626477541 | 0.0614657210401891 | 0.016548463356974 | 0.016548463356974 | 0.0319148936170213 | 0.0200945626477541 | 0.0118203309692671 | 0.016548463356974 | 0.0212765957446808 | 0.0153664302600473 | 0.0141843971631206 | 0.0260047281323877 | 0.0106382978723404 | 0.016548463356974 | 0.016548463356974 | 0.0295508274231678 | 0.0153664302600473 | 0.0200945626477541 | 0.0130023640661939 | 0.016548463356974 | 0.0106382978723404 | 0.0130023640661939 | 0.0106382978723404 |3
Supplement 2: Bactrial diversity in curcuma

## Slide 4
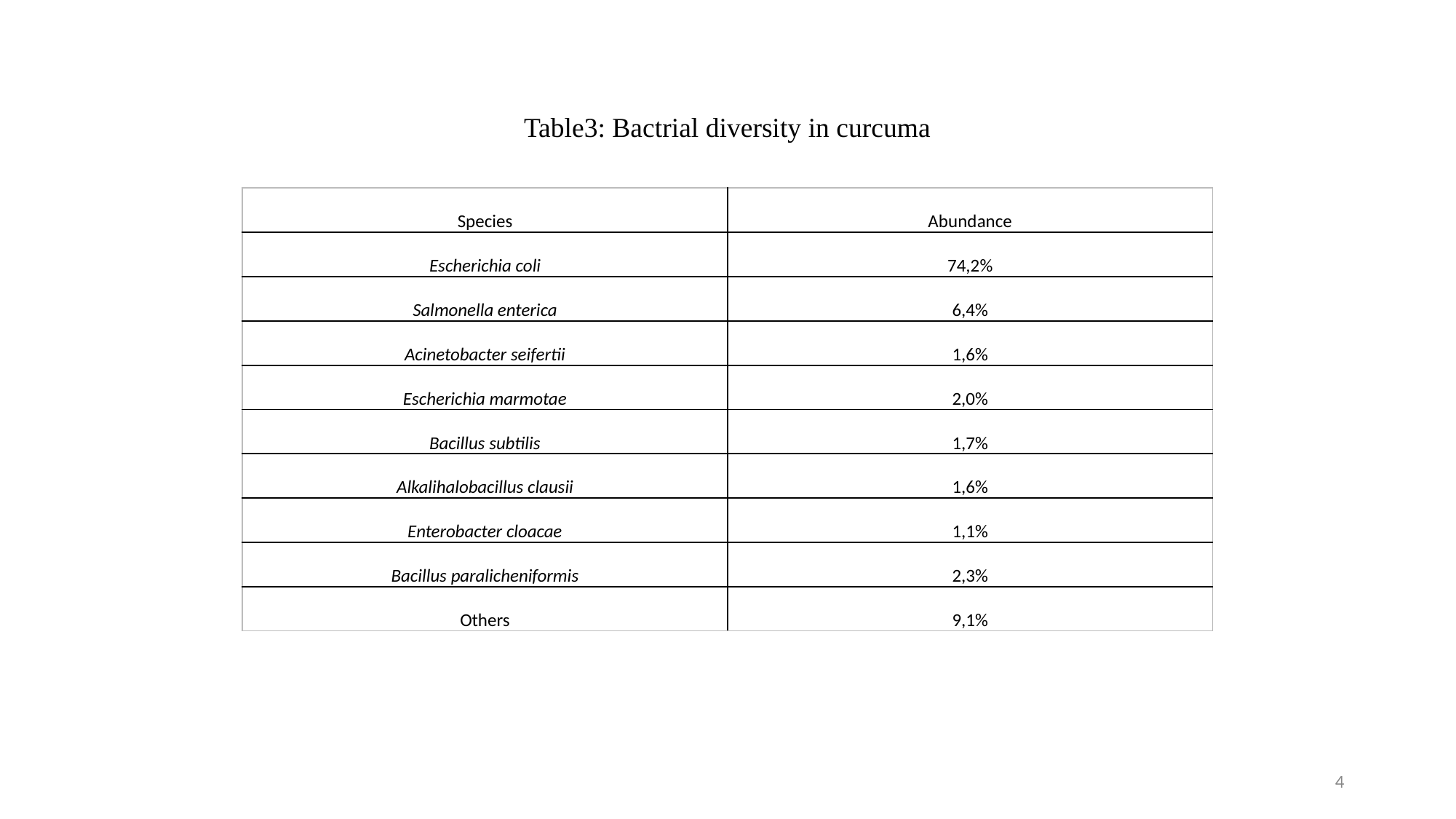

Table3: Bactrial diversity in curcuma
| Species | Abundance |
| --- | --- |
| Escherichia coli | 74,2% |
| Salmonella enterica | 6,4% |
| Acinetobacter seifertii | 1,6% |
| Escherichia marmotae | 2,0% |
| Bacillus subtilis | 1,7% |
| Alkalihalobacillus clausii | 1,6% |
| Enterobacter cloacae | 1,1% |
| Bacillus paralicheniformis | 2,3% |
| Others | 9,1% |
4

## Slide 5
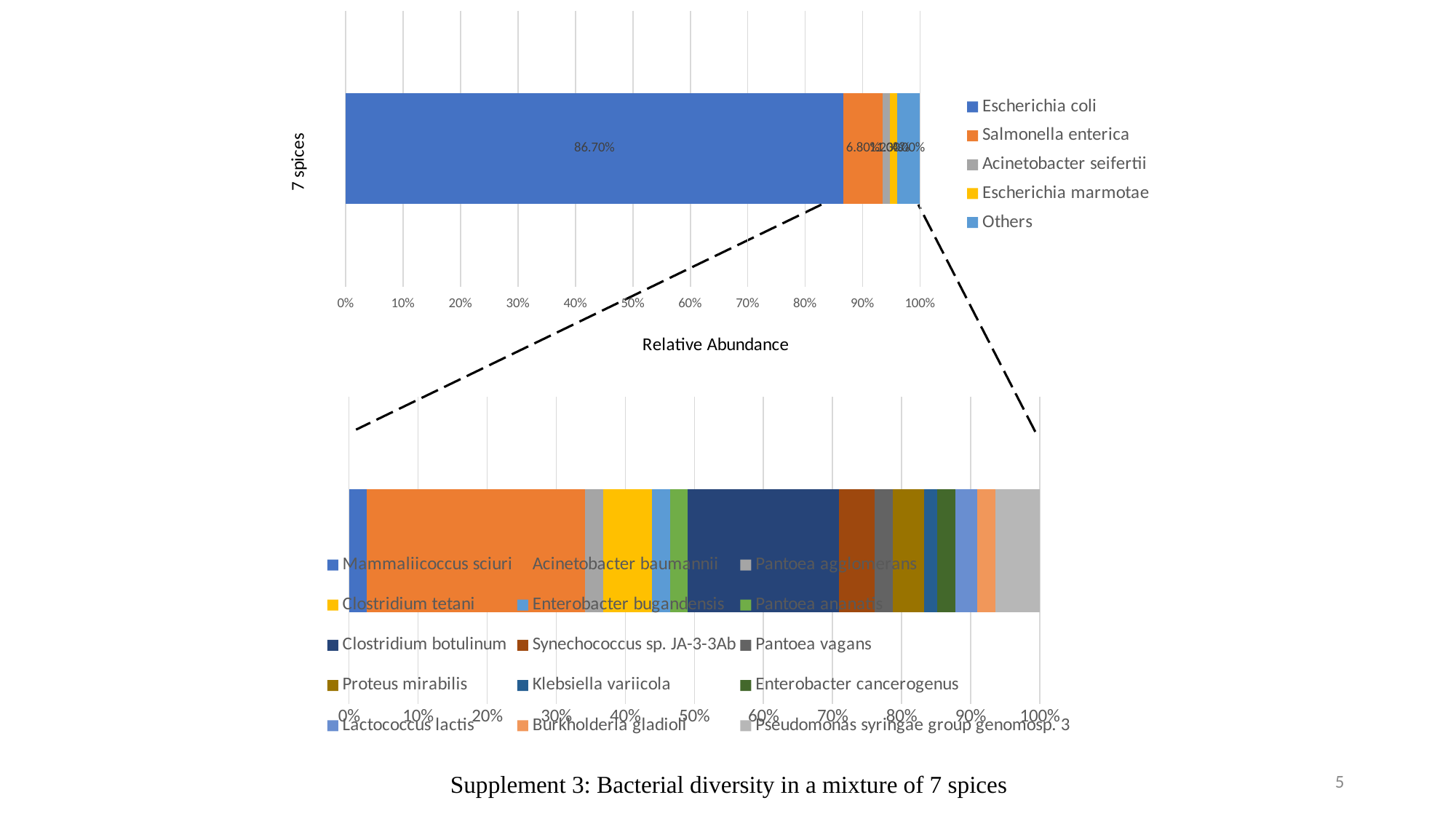

### Chart
| Category | Escherichia coli | Salmonella enterica | Acinetobacter seifertii | Escherichia marmotae | Others |
|---|---|---|---|---|---|
| relative abundance | 0.867 | 0.068 | 0.012 | 0.013 | 0.04 |
### Chart
| Category | | | | | | | | | | | | | | | |
|---|---|---|---|---|---|---|---|---|---|---|---|---|---|---|---|
| 8.6666666666666697E-2 | 0.0133333333333333 | 0.163333333333333 | 0.0133333333333333 | 0.0366666666666667 | 0.0133333333333333 | 0.0133333333333333 | 0.113333333333333 | 0.0266666666666667 | 0.0133333333333333 | 0.0233333333333333 | 0.01 | 0.0133333333333333 | 0.0166666666666667 | 0.0133333333333333 | 0.0333333333333333 |5
Supplement 3: Bacterial diversity in a mixture of 7 spices

## Slide 6
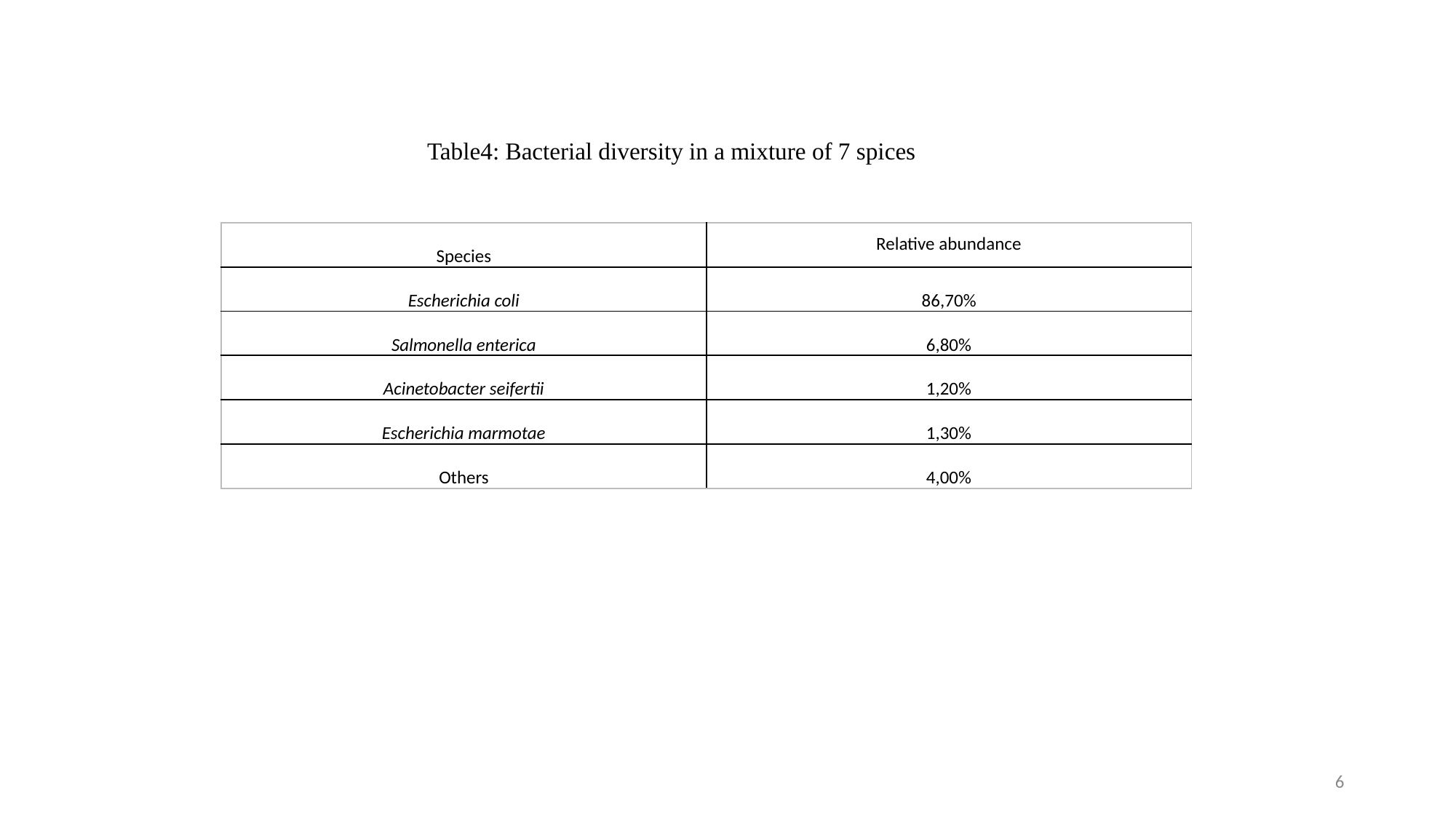

Table4: Bacterial diversity in a mixture of 7 spices
| Species | Relative abundance |
| --- | --- |
| Escherichia coli | 86,70% |
| Salmonella enterica | 6,80% |
| Acinetobacter seifertii | 1,20% |
| Escherichia marmotae | 1,30% |
| Others | 4,00% |
6

## Slide 7
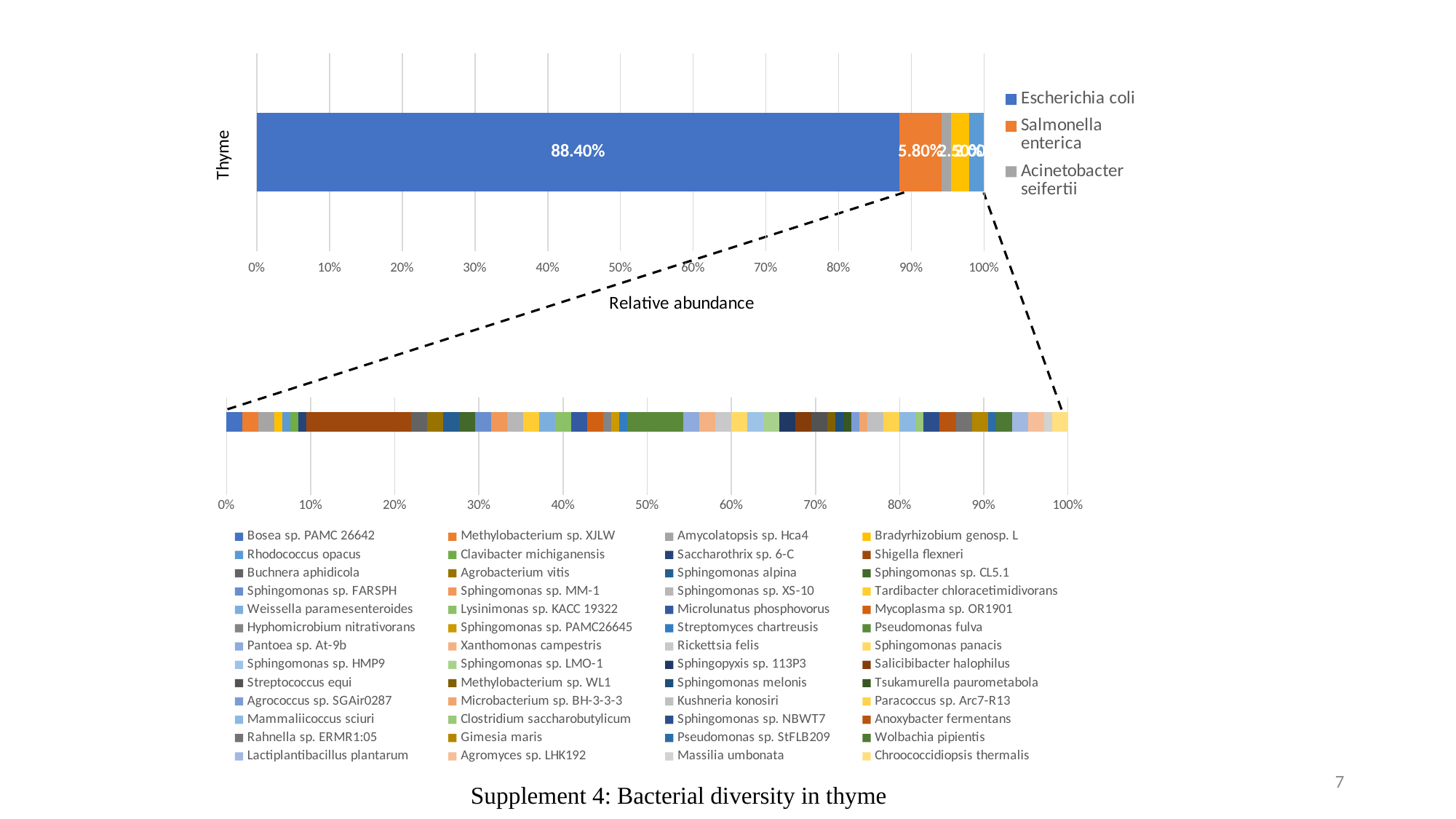

### Chart
| Category | Escherichia coli | Salmonella enterica | Acinetobacter seifertii | Escherichia marmotae | Others |
|---|---|---|---|---|---|
| Relative abundance | 0.884 | 0.058 | 0.013 | 0.025 | 0.02 |
### Chart
| Category | Bosea sp. PAMC 26642 | Methylobacterium sp. XJLW | Amycolatopsis sp. Hca4 | Bradyrhizobium genosp. L | Rhodococcus opacus | Clavibacter michiganensis | Saccharothrix sp. 6-C | Shigella flexneri | Buchnera aphidicola | Agrobacterium vitis | Sphingomonas alpina | Sphingomonas sp. CL5.1 | Sphingomonas sp. FARSPH | Sphingomonas sp. MM-1 | Sphingomonas sp. XS-10 | Tardibacter chloracetimidivorans | Weissella paramesenteroides | Lysinimonas sp. KACC 19322 | Microlunatus phosphovorus | Mycoplasma sp. OR1901 | Hyphomicrobium nitrativorans | Sphingomonas sp. PAMC26645 | Streptomyces chartreusis | Pseudomonas fulva | Pantoea sp. At-9b | Xanthomonas campestris | Rickettsia felis | Sphingomonas panacis | Sphingomonas sp. HMP9 | Sphingomonas sp. LMO-1 | Sphingopyxis sp. 113P3 | Salicibibacter halophilus | Streptococcus equi | Methylobacterium sp. WL1 | Sphingomonas melonis | Tsukamurella paurometabola | Agrococcus sp. SGAir0287 | Microbacterium sp. BH-3-3-3 | Kushneria konosiri | Paracoccus sp. Arc7-R13 | Mammaliicoccus sciuri | Clostridium saccharobutylicum | Sphingomonas sp. NBWT7 | Anoxybacter fermentans | Rahnella sp. ERMR1:05 | Gimesia maris | Pseudomonas sp. StFLB209 | Wolbachia pipientis | Lactiplantibacillus plantarum | Agromyces sp. LHK192 | Massilia umbonata | Chroococcidiopsis thermalis |
|---|---|---|---|---|---|---|---|---|---|---|---|---|---|---|---|---|---|---|---|---|---|---|---|---|---|---|---|---|---|---|---|---|---|---|---|---|---|---|---|---|---|---|---|---|---|---|---|---|---|---|---|---|7
Supplement 4: Bacterial diversity in thyme

## Slide 8
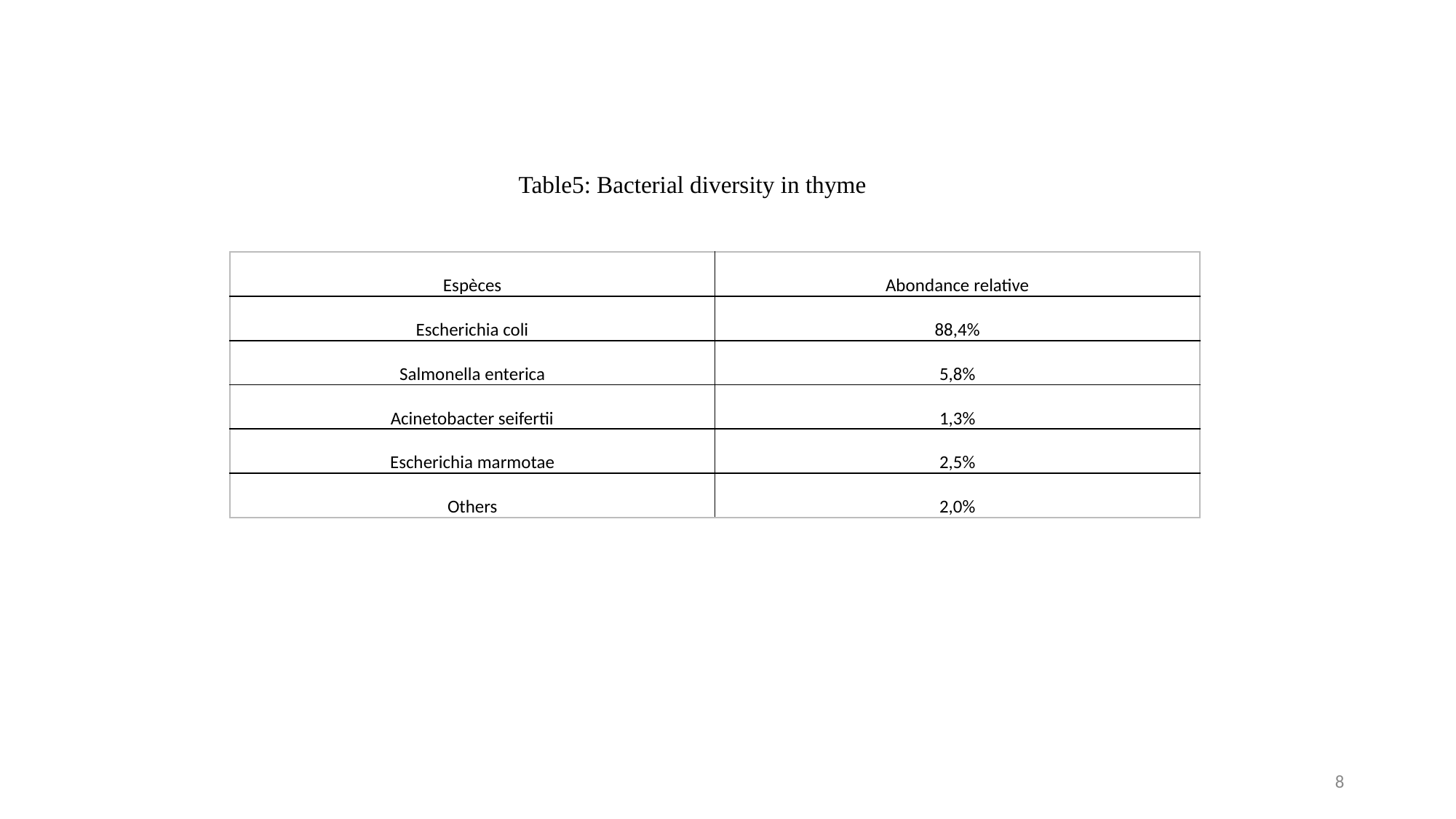

Table5: Bacterial diversity in thyme
| Espèces | Abondance relative |
| --- | --- |
| Escherichia coli | 88,4% |
| Salmonella enterica | 5,8% |
| Acinetobacter seifertii | 1,3% |
| Escherichia marmotae | 2,5% |
| Others | 2,0% |
8

## Slide 9
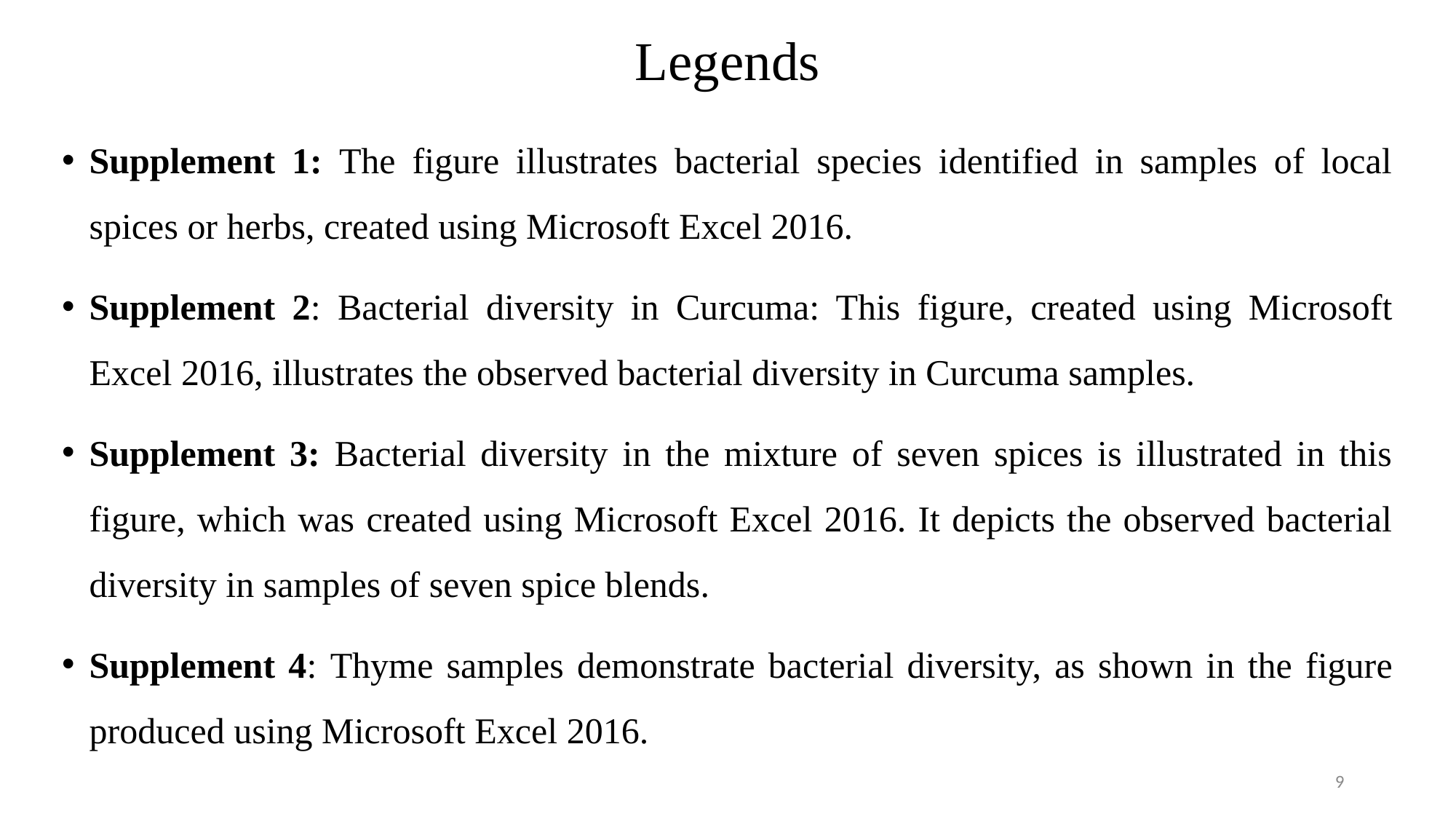

# Legends
Supplement 1: The figure illustrates bacterial species identified in samples of local spices or herbs, created using Microsoft Excel 2016.
Supplement 2: Bacterial diversity in Curcuma: This figure, created using Microsoft Excel 2016, illustrates the observed bacterial diversity in Curcuma samples.
Supplement 3: Bacterial diversity in the mixture of seven spices is illustrated in this figure, which was created using Microsoft Excel 2016. It depicts the observed bacterial diversity in samples of seven spice blends.
Supplement 4: Thyme samples demonstrate bacterial diversity, as shown in the figure produced using Microsoft Excel 2016.
9
